# Scaling gene expression for cell size control and senescence in *Saccharomyces cerevisiae*

**DOI:** 10.1007/s00294-020-01098-4

**Published:** 2020-11-05

**Authors:** Yuping Chen, Bruce Futcher

**Affiliations:** 1 Department of Chemical and Systems Biology, Stanford Medicine, Stanford, CA 94305-5174 USA; 2 Department of Microbiology and Immunology, Stony Brook University, Stony Brook, NY 11794-5222 USA

**Keywords:** Yeast, Cell size, Cell division, Size control, G1 S transition, Start

## Abstract

Cells divide with appropriate frequency by coupling division to growth—that is, cells divide only when they have grown sufficiently large. This process is poorly understood, but has been studied using cell size mutants. In principle, mutations affecting cell size could affect the mean size (“set-point” mutants), or they could affect the variability of sizes (“homeostasis” mutants). In practice, almost all known size mutants affect set-point, with little effect on size homeostasis. One model for size-dependent division depends on a size-dependent gene expression program: Activators of cell division are over-expressed at larger and larger sizes, while inhibitors are under-expressed. At sufficiently large size, activators overcome inhibitors, and the cell divides. Amounts of activators and inhibitors determine the set-point, but the gene expression program (the rate at which expression changes with cell size) determines the breadth of the size distribution (homeostasis). In this model, set-point mutants identify cell cycle activators and inhibitors, while homeostasis mutants identify regulators that couple expression of activators and inhibitors to size. We consider recent results suggesting that increased cell size causes senescence, and suggest that at very large sizes, an excess of DNA binding proteins leads to size induced senescence.

## Introduction

Cells couple division to growth. Cells must neither divide more than they grow (lest they shrink to death), nor grow more than they divide (lest they explode). At least for microbes, the coupling of division to growth seems to involve measurement of cell size. Cells have a “critical size” for commitment to division (Hartwell and Unger 1977; Johnston et al. 1977). Cells smaller than critical size grow, but do not divide, while cells larger than the critical size divide, and so become smaller. The requirement for a minimum size makes division dependent upon growth, and, together with division at larger sizes, maintains cells in a reasonably narrow size range. The cell size distribution is characteristic for a cell type in a given environment.

For this system, the cell must have a way of measuring size, so that the cell “knows” whether it has achieved critical size or not. Molecular biologists are familiar with mechanisms by which cells glean environmental information. The lac operon of *E. coli* is a mechanism for sensing lactose, while the Gal4/Gal80 system of the yeast *S. cerevisiae* is a mechanism for sensing galactose. In these and other cases, cells measure the presence of a small molecule (lactose, galactose) using a protein that binds tightly and specifically to it (lac repressor, Gal3). But, how does a sensing system for cell size work? What, exactly, is being measured, and how?

One way to approach this question has been to think about the geometry of growing cells (e.g., Fantes et al. 1975). For a spherical cell of diameter *D*, the cell’s surface area will be proportional to *D*^2^, while the volume will be proportional to *D*^3^. Thus, the changing ratios between diameter, surface area, and volume, and all cellular components that scale with these, could provide a molecular signal. For example, volume increases faster with growth than surface area, and a critical ratio of volume to surface area could send a signal. Or, for a cell in G1, DNA content is constant, but diameter, surface area, and volume are growing. So some critical ratio of diameter, surface area, or volume to DNA could send a signal. But precisely what molecular signals these might be is unclear.

The geneticist’s approach, pioneered by Paul Nurse using the yeast *Schizosaccharomyces pombe*, was to find mutants that alter cell size, and in that way directly discover genes controlling size, whatever their nature might be. Early screens found the *wee1* mutant in 1975 (Nurse 1975), the *wee2* mutant in 1978 (Thuriaux et al. 1978), and, in *S. cerevisiae*, the *WHI1* mutant in 1980 (Sudbery et al. 1980). For technical reasons, these screens focused on mutants with sizes smaller (“wee”-er) than wild-type.

The *S. pombe* Wee1 protein is a tyrosine kinase that inhibits the activity of the central cell cycle cyclin dependent kinase (CDK) Cdc2. Thus *wee1* has high Cdc2 kinase activity, and enters cell division “early”, in the sense that cells are still small. The *S. pombe wee2* mutant is an allele of *cdc2*^+^ itself, which likewise results in high Cdc2 activity and early cell division. *S. cerevisiae WHI1-1* encodes a stabilized form of a G1 cyclin now called Cln3, and also results in high activity of the cyclin dependent kinase (of the *S. cerevisae* cell cycle CDK, Cdc28), also causing early cell cycle entry. Thus, these cell size mutants target the CDK activity at very heart of the cell cycle machinery. In hindsight, we would argue that many of the cell size mutants were more successful in promoting understanding of the basic mechanisms of cell cycle entry than they were for understanding cell size.

The study of these and other cell size mutants has continued for more than four decades, and although much progress has been made (Amodeo and Skotheim 2016; Ginzberg et al. 2015; Heldt et al. 2018; Jorgensen and Tyers 2004; Schmoller and Skotheim 2015; Turner et al. 2012; Zatulovskiy and Skotheim 2020), still there is no clear understanding of how size control works. Part of the issue is that cell size control constitutes two separate sub-problems, which are often conflated or confused.

**Fig. 1:**
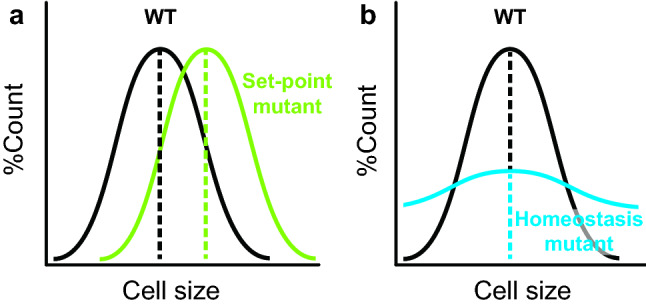
Set-point mutant vs homeostasis mutant. **a** A set-point mutant has an altered mean cell size, but the width of the size distribution is wild-type (when measured as the coefficient of variation, to normalize for the different means). **b** A homeostasis mutant has an altered width of the size distribution (when measured as coefficient of variation), but may have a wild-type mean cell size

The first problem we call the “set-point” problem (Fig. 1a), and it is the problem of how the critical size is set. For instance, in *S. cerevisiae*, the mean critical size is roughly 35 femtoLiters (fL), while some *whi* mutants enter the cell cycle at about 25 fL (e.g., *CLN3-1*, *whi5*). Thus, the *CLN3-1* and *whi5* mutants have a defect in the critical size “set-point”. To our knowledge, all screens for cell size mutants (Dungrawala et al. 2012; Jorgensen et al. 2002; Navarro and Nurse 2012; Soifer and Barkai 2014; Zhang et al. 2002) have screened for mutants with an altered set-point.

But there is also a second, conceptually different problem we call the “homeostasis” problem (Fig. 1b). This has to do with the variation of cell size around the mean. One can imagine two different cell types, both with a mean critical size of 35 fL, but where one cell type, with strong cell size control, might have a narrow size distribution around that mean, while another cell type, with sloppy size control, might have a wide distribution around that mean. Depending on the molecular nature of size control, “set-point” and “homeostasis” might be controlled independently, by different mechanisms.

The “set-point” problem is widely recognized, partly because it is defined by the existence of cell size mutants with altered mean sizes. The “homeostasis” problem is less obvious, and has been addressed most clearly in the *S. pombe* system. One example is Sveiczer et al. (1996) [with a follow-up from Sveiczer et al. (1999)] (Sveiczer et al. 1996, 1999), where the coefficient of variation (i.e., the standard deviation divided by the mean, the normalized variation) of the cell size distribution was measured for several cell size mutants. (Larger cells will always have more size variance than smaller cells, just as elephants have more size variance, in kilograms, than mice. It is essential that homeostasis be studied using normalized variance, i.e., the coefficient of variation.) Mutants in *wee1*, originally isolated as a strong set-point mutant, had a coefficient of variation larger than wild-type. The coefficient of variation was even larger in a *wee1-50* *cdc25*∆ double mutant. (Cdc25 is normally essential, because it removes a CDK-inhibitory phosphate placed by Wee1. But it is dispensable in a *wee1* mutant.) Thus, *wee1* mutations simultaneously affect both set-point and homeostasis, which is what one might imagine if *wee1* mutants were generally defective in size control.

Wood and Nurse (2013) examined size homeostasis in several *S. pombe* size mutants using two assays. One assay measured the coefficient of variation of cell size, as above, while the other measured, for individual cells, the relationship between cell size at birth, and the size added before division. With good size control, cells born smaller add more size before division. For the size mutants *pom1* and *nif1*, little evidence was found for any homeostasis defect. But [partly confirming results of Sveiczer et al. (1996)], in a *cdc13-L-cdc2AF* strain [where the CDK Cdc2 cannot be regulated by Wee1 or Cdc25, similar to the *wee1-50* *cdc25*∆ strain of Sveizer et al. (1996)], cell size did have a somewhat higher coefficient of variation than wild-type. The effect was moderate, and clearly the mutant strain had substantial remaining size control. Furthermore, by the second assay (the anti-correlation between size at birth, and size added), the *cdc13-L-cdc2AF* strain had essentially wild-type size homeostasis. Overall, although *S. pombe* set-point mutants working through tyrosine phosphorylation of Cdc2 (i.e., Wee1/Cdc25) have some defect in homeostasis, there is a great deal of homeostatic control left over even when control of this phosphorylation is removed.

In *S. cerevisiae*, set-point has been studied almost to the complete exclusion of homeostasis. All screens for cell size mutants have looked for mutants with an altered mean cell size, i.e., an altered set-point. In a few cases, where the coefficients of variation of size in set-point mutants have been measured, workers have been surprised to find little or no defect in homeostasis (Di Talia et al. 2009; Schneider et al. 2004; Wang et al. 2009).

Recently, we systematically examined the *S. cerevisiae* set-point mutants (Chen et al. 2020). All 32 known set-point mutants were assayed using a Coulter Channelyzer for their co-efficients of variation. Remarkably (at least to us), 30 of 32 mutants had no detectable defect in cell size homeostasis (within experimental error), while two (*cdh1* and *spt4*) had modest defects.

This is a striking result. These set-point mutants are thought to define the cell’s mechanisms of controlling size, and indeed several of them (*cln3*, *whi5*, *bck2*) work at the heart of the cell cycle machinery and certainly do affect both cell size and the timing of the G1/S transition. And yet, mutants without these genes control the variability in their cell sizes just as effectively as wild-type (albeit to an altered mean).

**Fig. 2:**
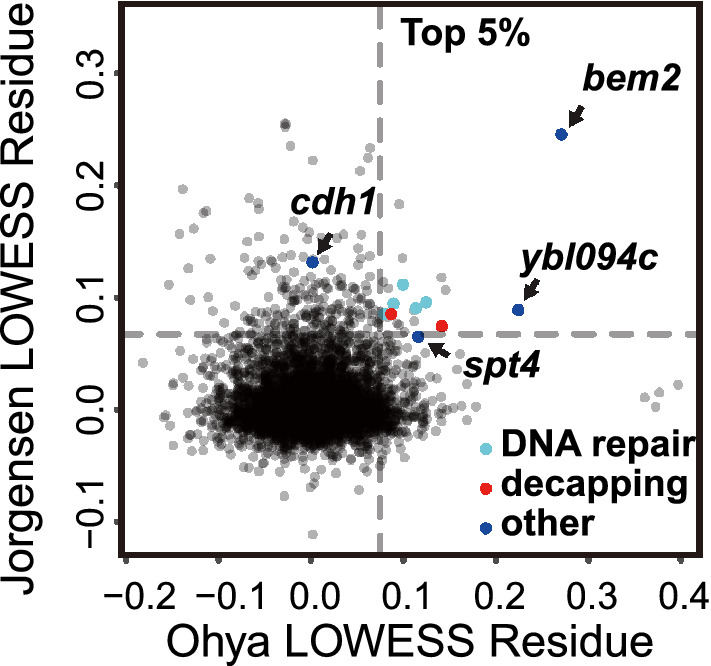
Possible homeostasis mutants from previous genome-wide measurements. Cell size coefficients of variation (CV) from two genome wide screens (Jorgensen et al. 2002; Ohya et al. 2005). Large positive values indicate large CVs. *Y*-axis: Jorgensen et al. *X*-axis: cell size of mother cells with a small to medium bud (parameter IDs: CCV111_A1B, C111_A1B) from Ohya et al. Both axes show the LOWESS (locally weighted scatterplot smoothing) (*f* = 0.1) residues from CV vs mean plot. Dashed lines indicate the top 5%. Light blue: DNA repair mutants. Red: mRNA decapping mutants. Navy blue: four mutants with large CV measured by Chen et al. (2020). Grey (lower right): four mutants with large CV only in the Ohya dataset are *rpl34b*, *rsr1*, *ygr151c* (ORF overlaps with *BUD1*) and *bud2*

In genetics, you get what you select for, and in the cell size field, workers have selected for set-point mutants. Do undiscovered homeostasis mutants exist? No direct screens have been done. However, Ohya et al. and Jorgensen et al. made cell size and variability measurements of the yeast deletion set (Jorgensen et al. 2002; Ohya et al. 2005). We searched these genome-wide datasets for mutants with high variability in both studies (Fig. 2). One mutant with such variability is *bem2* (Bud EMergence 2), but our analysis by time-lapse microscopy shows, consistent with previous results, that *bem2* has abnormal bud morphology. We find a stochastic defect in nuclear division, such that the daughter nucleus sometimes does not segregate into the new bud, leading to a population with mixed ploidies (including a ploidy of zero). We believe this budding and division defect is responsible for the large co-efficient of variation, and we see no evidence for a defect in size-sensing. Another mutant with high variability in both datasets is the dubious ORF *YBL094C*. However, tetrad analysis showed that the high CV phenotype of this particular mutant from the deletion set does not co-segregate with the *ybl094C* deletion. Instead, sequencing showed that the high CV phenotype co-segregated with a stop codon mutation (TCA to TAA) at Ser248 of Bem2. Thus, both mutants with very high size variability are alleles of *BEM2*.

There were 18 other mutants with a CV in the top 5% in both studies: *shp1*, *cyt1*, *dcs1*, *rad54*, *yjr011c*, *rpl13b*, *ybr225w*, *isa1*, *rad51*, *met5*, *rps19b*, *hcr1*, *mms1*, *rai1*, *ygl218w*, *yjr146w*, *slx5* and* mrpl17*. These include five genes involved in DNA repair/genome integrity: *rad54*, *rad51*, *yjr011c*, *mms1*, and *slx5*. Based on experience with such mutants, we believe they have a high CV, because some cells in the population sustain incidental DNA damage during replication; these arrest at a damage checkpoint but grow in size; and this arrest-with-growth gives a high CV.

The genes *shp1*, *rpl13b*, *rps19b*, and *hcr1* (and *cdh1*) affect protein production and degradation. These genes could relate to differential scaling of cell cycle activators and inhibitors at the protein level and thus size homeostasis as discussed below.

Finally, there were two genes involved in mRNA decapping and degradation: *rai1* and *dcs1*. The mRNA degradation gene *xrn1* narrowly missed being top 5% in both studies. Because the rate of decapping and degradation is critical for determining the half-life of an mRNA, these mutants could lead to abnormally long mRNA half-lives, which could be connected to size homeostasis (see below).

However, over all, effects on homeostasis were small, and we believe insufficient to explain the good homeostasis of wild-type cells. It is possible that strong homeostasis mutants exist, but are inviable.

One recent idea for how size control might work is the Whi5 dilution model of Schmoller et al. (2015). In this model, Whi5, a cell cycle inhibitor, is pitted against Cln3, a cell cycle activator. In small cells, the concentration of Whi5 is relatively high, and so inhibition predominates. But as cells grow, Whi5 is diluted and diluted, until at critical size it can no longer keep Cln3 in check, and cell cycle entry occurs. Although this model has many advantages and is surely part of the truth, it seems inadequate as complete explanation, because a *whi5* null is alive, with small cells, but with about the same coefficient of variation for size as wild-type (Barber et al. 2020; Chen et al. 2020). That is, even in the absence of Whi5, cells can efficiently couple division to growth, controlling their size variation (around a different set-point, of course).

Keifenheim et al. (2017) proposed a related model invoking size-dependent expression of the cell cycle activator Cdc25 (in fission yeast). In this model, larger and larger cells express higher and higher concentrations of Cdc25, thus activating division at some sufficiently large size. But again, although very attractive as a partial explanation, it is inadequate as a complete explanation, because (as discussed above) mutants, where Cdc25 is absent or ineffective retain a great deal of homeostatic control (Wood and Nurse 2013).

**Fig. 3:**
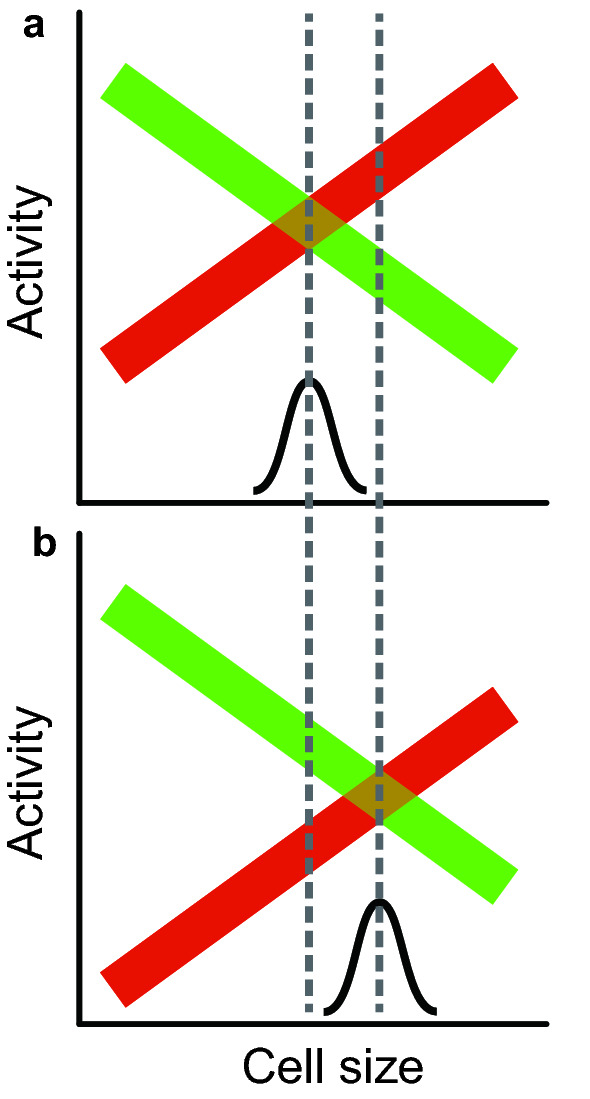
Model for a size-dependent cell cycle, and for a set-point mutant. **a** In small cells, a set of inhibitors (green) predominate over activators (red). But as cells grow, the concentration of activators increases, and concentration of inhibitors decreases, until at critical size activators predominate, and trigger cell cycle entry. The variability in cell sizes is determined by the stochastic overlap between activator and inhibitor concentrations. **b** A set-point mutant. In this mutant (e.g., *cln3*), one activator is entirely missing, so the whole activator curve is shifted down, and critical size (the set-point, where the activator line crosses the inhibitor line) is shifted to the right, to larger sizes. But the slopes of the remaining activators and inhibitors do not change, and so size variability does not change

**Fig. 4:**
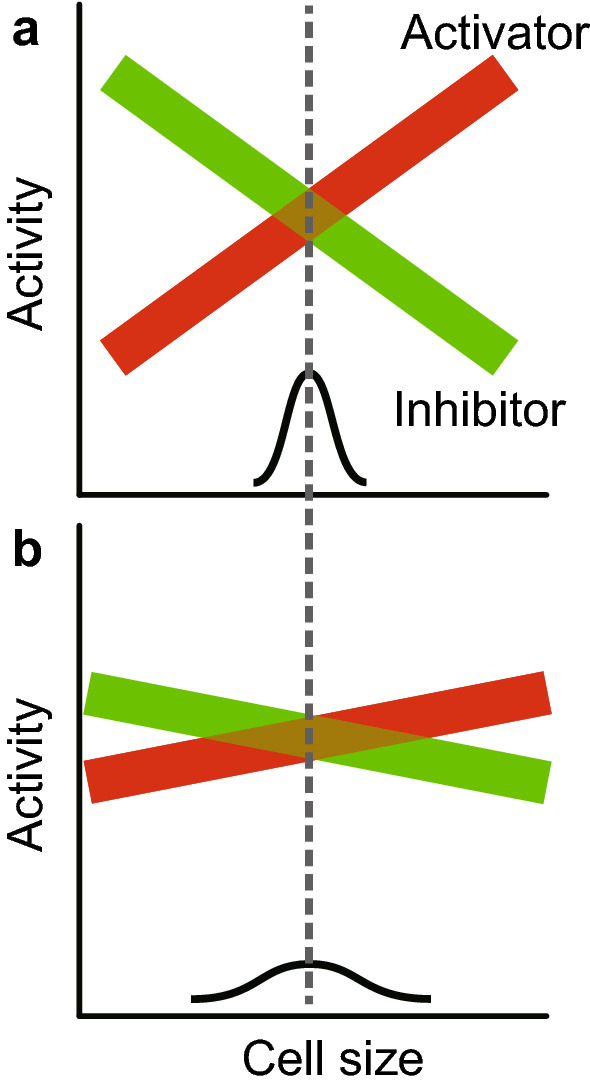
Model for a homeostasis mutant. **a** Wild-type. **b**. Homeostasis mutant. The size-dependence of gene expression for both activators and inhibitors has been decreased, so the slopes of activity vs size are now flatter than WT. There is a greater region of stochastic overlap, so size variability is larger. Any mutant causing a flattening of either slope (i.e., activators or inhibitors) will be a homeostasis mutant

We considered a more general form of these models, where many cell cycle inhibitors are pitted against many activators (Chen et al. 2020). We hypothesize, like Schmoller et al. or Keifenheim et al. that as cells grow in size, inhibitors tend to drop in concentration, while activators tend to increase, until, at critical size, activators triumph over inhibitors, and initiate the cycle (Figs. 3, 4) (we temporarily leave aside the problem of how these changes in concentration might occur). Testing this experimentally involves a few tricks. First, although the ultimate activators and inhibitors are proteins, mRNA amounts are generally excellent proxies for protein amounts (Newman et al. 2006), and are easy to measure using RNA-Seq. Second, one wants to look purely at changes in size, without changes in cell cycle state. Therefore, we isolated small G1-phase daughter cells using elutriation, and blocked cell cycle progress using a very specific CDK inhibitor. Thus cells would grow in size and protein content, but would remain in “early” (i.e., low CDK) G1 from the cell cycle point of view. Third, if all ~ 6000 genes were considered, inevitably some would seem to increase or decrease in concentration just because of stochastic noise. This would present a serious multiple hypothesis testing statistical problem. To circumvent this, we pre-chose (i.e., before looking at data) eight classic cell cycle activators and eight cell cycle inhibitors, and limited statistical analysis to these 16 (we imagine the total number of relevant activators and inhibitors might be even larger). Our specific hypothesis was that mRNA concentrations for the activators would increase with cell size, while those for the inhibitors would decrease.

Thus, we obtained small G1 cells, blocked them in G1 phase, let them grow to larger and larger sizes without cell cycle progress, took samples as size increased, and measured amounts of mRNA for every gene. This yielded a curve of gene expression as a function of cell size (Chen et al. 2020).

Overall (that is, over all ~ 6000 genes), gene expression increases almost exactly in proportion to cell size, so that for the vast majority of genes, mRNA concentration stays constant within the physiological cell size range. This is expected, and has been shown previously in *S. pombe* (Zhurinsky et al. 2010). However, for the eight pre-chosen activators, seven increased in abundance faster than the cells increased in size. That is, they increased in concentration as cells grew. We call this “super-scaling”. In contrast, for the eight pre-chosen inhibitors, all eight increased in abundance more slowly than the cells grew—that is, they decreased in concentration. We call this “sub-scaling”. The *p *value for the difference between activators and inhibitors was about 0.0006, and replicated over three experiments.

Thus, at small size, inhibitors predominate, preventing cell cycle progress. With growth, activators increase relative to inhibitors, until at some larger size activators predominate, triggering cell cycle entry (Fig. 3).

Activators increasing in concentration with cell size, and inhibitors decreasing in concentration, obviously would explain size-dependent division and size control. But what could be the mechanism of such size-dependent changes? At present, one can only speculate, so this remains a weak point of the model. We suggest two mechanisms for size-dependent gene expression (Chen et al. 2020). The clearest depends on the fact that DNA amounts stay constant through G1. Thus, as a G1-phase cell grows, the ratio of cell mass and protein to DNA increases steadily. One could imagine a co-operative transcription factor. As the cell grows, more and more of the transcription factor would be made, but the amount of target DNA would remain constant. So, increasing amounts of this co-operative transcription factor would be available to bind upstream of target genes, turning them on in a size-dependent way. Similarly, inhibitors could be controlled by a co-operative repressor, which would decrease gene expression in a size-dependent way.

The model of size-dependent expression of multiple cell cycle activators and inhibitors has several advantages. First, it allows multiple regulators, perhaps sensing different aspects of growth and environment, to partake in size control. Second, it allows the network of regulators to drift and change in different species, as required by their lifestyles. An issue with current models, which generally focus on one specific regulator, is that the regulator is often not conserved (e.g., *WHI5* or *CLN3*), or, if conserved, not always very important for size control (e.g., *cdc25* in *S. pombe* vs *S. cerevisiae*) in other species.

A third advantage is it explains the key point of why mutations in the activators or inhibitors change set-point but not homeostasis. The activators and inhibitors are the effectors of cell size, and their presence or absence will change the set-point (Fig. 3). But, crucially, they are not regulating the size-dependence of expression—they do not change the slope of the size-dependent expression. Homeostasis mutants would arise from changes that alter the way activators and inhibitors are expressed as a function of size. That is, homeostasis mutants would change the slope of activator/inhibitor expression. For example, in *S. cerevisiae*, *whi5* or *cln3* mutants change set-point. But to produce a change in homeostasis, a completely different kind of mutant is needed, which would change the degree of sub-scaling of *WHI5*, or the degree of super-scaling of *CLN3* (Fig. 4). That is, while mutating *WHI5* or *CLN3* changes set-point, mutating regulators of *WHI5* or *CLN3* would change homeostasis.

Motivated by this idea, we swapped the open reading frames of *CLN2* and *WHI5*, placing *CLN2* under control of the *WHI5* promoter, and vice versa. The idea is that now *CLN2* (driven by the *WHI5* promoter) might sub-scale (decrease concentration with size), while *WHI5* (driven by the *CLN2* promoter) might super-scale (increase concentration with size). Of course there are other activators and inhibitors behaving in a wild-type way, but nevertheless the aberrant, opposite-to-normal scaling of these two powerful effectors could produce poor homeostasis. And indeed, the “swap” mutant had a higher co-efficient of variation than the wild-type, or than individual or combined deletion mutants (Chen et al. 2020). This supports the model, suggesting that it is the size-dependent expression of activators and inhibitors that gives size homeostasis.

### Cell size and aging

As described above, with growth, the vast majority of proteins increase in proportion to cell mass. But some of these proteins bind DNA—and at least in G1 phase, the amount of DNA is constant. Thus, in general, one expects that as a G1-phase cell grows, the ratio of DNA-binding protein (of all kinds) to DNA also grows. On the one hand, this can be the basis of a model for size-dependent gene expression (see above), in which increasing amounts of transcriptional activator per target gene drives super-scaling while increasing amounts of transcriptional repressor per target gene drives sub-scaling. However, leaving aside these cases, it seems almost inevitable that for DNA binding proteins in general, there will be a higher ratio of protein-to-DNA for larger cells than for smaller cells. Cells in the physiological size range may be able to cope with this, or may find it useful for size signaling.

**Fig. 5:**
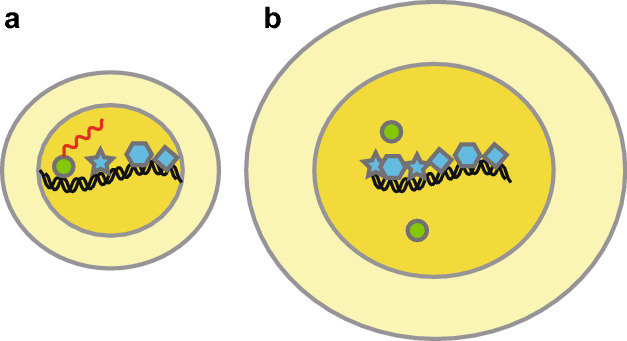
Model for size-induced senescence (SIS). **a** A normal cell. In the physiological size range, the amount of DNA-binding protein is well-matched to the amount of DNA. Transcription is normal (nascent mRNA in red). **b** A large cell. At large sizes, a larger amount of every protein is (almost unavoidably) made. However, since DNA amounts are independent of cell size, the ratio of DNA binding protein to DNA becomes large, and at extreme sizes causes misregulation of gene expression

But possibly if cells were to continue enlarging past the normal size range, the ever increasing DNA-binding-protein-to-DNA ratio might begin to have pathological effects. DNA might be coated with DNA binding proteins at a density that obscures normal gene expression (Fig. 5). This could interfere with cell division—a process highly dependent on regulated expression of hundreds of genes. Thus large cells could become locked into a state, where they are slow to divide, because a high protein-to-DNA-ratio interferes with gene expression—and the ensuing increase in cell size worsens the gene expression problem (Fig. 5). In this way, large cells might become larger cells, and eventually become permanently incapable of division, because of an irreversible problem of too high a protein-to-DNA ratio. One would call such an inability to divide “senescence”, and the phenomenon could be called “size-induced senescence” (SIS). Indeed, Amon et al. have recently shown that for both yeast and mammalian cells, large cell size as such may be a cause of senescence (Neurohr et al. 2019).

Many observations are supportive of the idea that increased cell size could cause senescence. To begin with the obvious, cells deemed “senescent” are invariably large. Oncogene-induced senescence comprises various treatments, for example over-expression of an oncogene such as activated K-ras, that leads to senescence (i.e., an inability to divide) and also leads to an increased cell size. Which is cause and which is effect is unclear. Similarly, upregulation of CDK inhibitors, or treatment with DNA damaging agents, can cause temporary cell cycle arrest, increased cell size, and, eventually, senescence. We feel the regulation of cell cycle by size-dependent gene expression, and size-induced senescence, could be related phenomena.
